# Investigation of a Camera-Based Contactless Pulse Oximeter with Time-Division Multiplex Illumination Applied on Piglets for Neonatological Applications

**DOI:** 10.3390/bios14090437

**Published:** 2024-09-09

**Authors:** René Thull, Sybelle Goedicke-Fritz, Daniel Schmiech, Aly Marnach, Simon Müller, Christina Körbel, Matthias W. Laschke, Erol Tutdibi, Nasenien Nourkami-Tutdibi, Elisabeth Kaiser, Regine Weber, Michael Zemlin, Andreas R. Diewald

**Affiliations:** 1 Laboratory of Applied Radar Technology and Optical Systems (LaROS), Trier University of Applied Sciences, Schneidershof, 54293 Trier, Germanys.mueller@et.hochschule-trier.de (S.M.); 2 Department of General Pediatrics and Neonatology, Saarland University, Campus Homburg, 66421 Homburg, Germany; sybelle.goedicke-fritz@uks.eu (S.G.-F.); erol.tutdibi@uks.eu (E.T.); nasenien.nourkami@uks.eu (N.N.-T.); elisabeth.kaiser@uks.eu (E.K.); regine.weber@uks.eu (R.W.); 3 Institute for Clinical and Experimental Surgery, Saarland University, 66421 Homburg, Germany; christina.koerbel@uks.eu (C.K.); matthias.laschke@uks.eu (M.W.L.)

**Keywords:** optical sensors, nonlinear dynamical systems, image sensors, biomedical signal processing, biomedical monitoring, in vivo, neonatology

## Abstract

(1) Objective: This study aims to lay a foundation for noncontact intensive care monitoring of premature babies. (2) Methods: Arterial oxygen saturation and heart rate were measured using a monochrome camera and time-division multiplex controlled lighting at three different wavelengths (660 nm, 810 nm and 940 nm) on a piglet model. (3) Results: Using this camera system and our newly designed algorithm for further analysis, the detection of a heartbeat and the calculation of oxygen saturation were evaluated. In motionless individuals, heartbeat and respiration were separated clearly during light breathing and with only minor intervention. In this case, the mean difference between noncontact and contact saturation measurements was 0.7% (RMSE = 3.8%, MAE = 2.93%). (4) Conclusions: The new sensor was proven effective under ideal animal experimental conditions. The results allow a systematic improvement for the further development of contactless vital sign monitoring systems. The results presented here are a major step towards the development of an incubator with noncontact sensor systems for use in the neonatal intensive care unit.

## 1. Introduction

Almost 10% of all children are at increased risk for long-term sequelae due to preterm birth [1,2]. The medical care required for the survival of premature babies often lasts for months, and constant monitoring using conventional techniques can cause pain and skin irritation [3,4,5]. In the context of intensive care, it is necessary to observe vital parameters regularly over a longer period of time. In the search for better ways to monitor such vulnerable infants without causing distress, contactless measurement methods are becoming increasingly important [4,6]. Pulse oximetry (PO) is a standard technique used to continuously measure the proportion of hemoglobin molecules that are loaded with oxygen in arterial blood [7,8]. Common pulse oximeters (PO-meters) are poorly suited to preterm neonates since they usually require a circular bandage for the sensor and a wire with a diameter of approximately 4 mm. The circular bandage can disturb the perfusion of extremities and cause skin lesions during removal, and the wire can cause entanglements or decubitus ulcers when the infant moves. Moreover, motion artifacts are very frequent [5,8,9,10,11]. The aim of this project is to close the gap in vital monitoring of premature infants by developing a sensor that measures the infant’s vital signs (heartbeat and oxygen saturation) by means of a camera, without requiring any skin contact.

In contrast to wearable technologies, which work with wireless technologies for communication [12,13,14], remote sensors work without any contact to the patient. A very good overview of cable-based and contactless sensors is given in a review paper from 2024 [15]. In the review, contactless remote photoplethysmography (rPPG) sensor systems were reviewed, but the reviewed technology was without any active illumination, and only a few publications focused on neonate intensive care rather than on measurements in adults. A very interesting rPPG sensor approach for neonate intensive care was presented in [14,16,17]. There, two independent camera systems with different optical filters in the near-infrared for each camera were applied for heartbeat detection and SpO_2_ measurement. In our publication, we demonstrate the functionality of an rPPG system but with active illumination in the optical near-infrared (NIR) to get rid of passive ambient light. This sensor system was applied on an animal model to pave the way for its use in neonatal intensive care units (NICUs).

## 2. Materials and Methods

**Figure 1:**
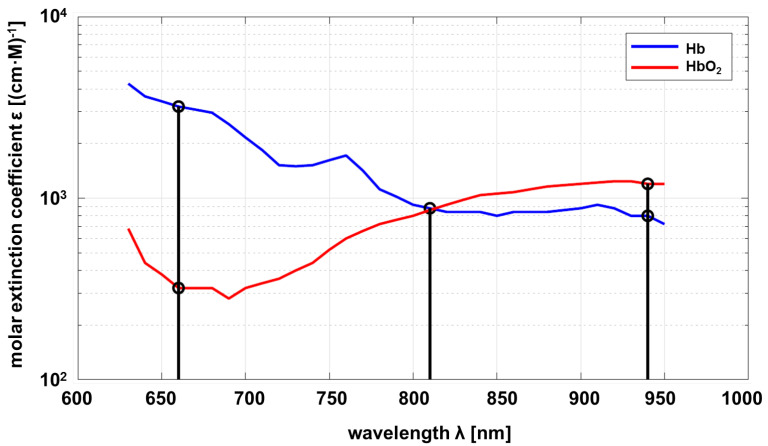
Molar absorption spectrum of hemoglobin and oxyhemoglobin based on Prahl [18].

According to the Beer–Lambert law, the optical properties of a substance to be investigated must be known as a function of wavelength. In this project, the relevant substance is circulating blood, in particular hemoglobin, which is responsible for oxygen transport. As a consequence, the properties of deoxyhemoglobin (Hb) and oxyhemoglobin (HbO_2_) are of interest. The molar absorption of Hb and HbO_2_ depending on the wavelength is shown in Figure 1.

Common pulse oximeters use two wavelengths at approximately $\lambda_{1}=660$ nm and $\lambda_{3}=940$ nm. The sensor developed within this project uses an additional wavelength at $\lambda_{2}=810$ nm as proposed by Wieringa et al. [19]. The maximum difference in absorption of Hb compared to HbO_2_ is at $\lambda_{1}$, and vice versa at $\lambda_{3}$. Both substances show the same absorption at $\lambda_{2}$ (adiabatic point). The linear independence of the transmission at different wavelengths allows a statement about the composition ratio of Hb and HbO_2_ if measurements at different wavelengths are carried out. The mathematical principles are described below [20,21].

### 2.1. Pulse Oximetry

The calculation of the oxygen saturation of the blood is based on the principles of Beer–Lambert’s law (Equation (1)), which describes the absorbance of blood, in particular hemoglobin. This depends on the density *c*, (decadic) extinction coefficient ε and path length *l* at which the light passes through an artery. $I_{\lambda }^{i}$ means the intensity or power of the incident light and $I_{\lambda }^{t}$ means the intensity of transmitted light escaping from the measuring medium.

(1) $$A_{\lambda }=\log_{10}\left(\frac{I_{\lambda }^{i}}{I_{\lambda }^{t}}\right)=\varepsilon_{\lambda }\cdot c\cdot l$$

It can also be written in exponential form for the transmittance with extinction coefficient $\varepsilon_{\lambda }^{n}$ (natural base) written in the following as ε.

(2) $$I_{\lambda }^{t}=\left(I_{\lambda }^{i}-I_{\lambda }^{r}\right)\cdot e^{\varepsilon \cdot c\cdot l}$$

**Figure 2:**
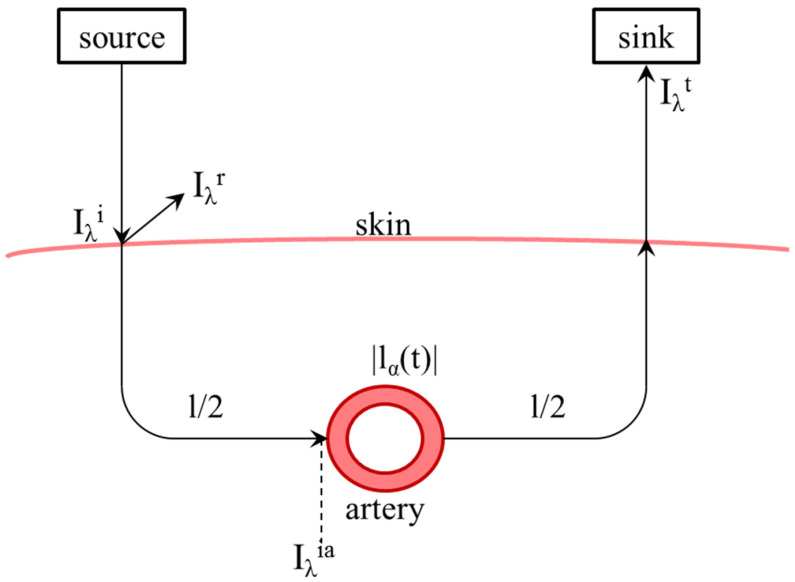
Schematic representation of the light path from the transmitter through the skin and back to the receiver including remission and scattering as well as the path length *l* and the time-dependent path length l(t) in the artery. $I_{\lambda }^{i}$ means the intensity or power of the incident light and $I_{\lambda }^{t}$ means the intensity of the light escaping from the measuring medium. $I_{\lambda }^{r}$ means the power of reflected light.

This equation is intended to describe one measuring medium with a constant path length. For real application to measure oxygen saturation, different attenuations caused by skin, bone and other nonpulsating elements lead to a constant part *l*, as well as in an alternating $l_{a}\left(t\right)$ caused by the pulse wave in arteries and vessels, shown in Figure 2.

(3) $$\begin{array}{cc} I_{\lambda }^{ia} & =\left(I_{\lambda }^{i}-I_{\lambda }^{r}\right)\cdot e^{-\varepsilon \cdot c\cdot l} \end{array}$$

(4) $$\begin{array}{cc} I_{\lambda }^{t}\left(t\right) & =I_{\lambda }^{ia}\cdot e^{-\varepsilon_{a}\cdot c_{a}\cdot l_{a}\left(t\right)} \end{array}$$

so it follows:

(5) $$\begin{array}{cc} I_{\lambda }^{t}\left(t\right) & =\left(I_{\lambda }^{i}-I_{\lambda }^{r}\right)\cdot e^{-(\varepsilon \cdot c\cdot l+\varepsilon_{a}\cdot c_{a}\cdot l_{a}\left(t\right))} \end{array}$$

(6) $$\begin{array}{cc} I_{\lambda }^{t}\left(t\right) & =\left(I_{\lambda }^{i}-I_{\lambda }^{r}\right)\cdot e^{-(\alpha \cdot l+\alpha_{a}\cdot l_{a}\left(t\right))} \end{array}$$

The transmission $T_{\lambda ,a}\left(t\right)$ is the ratio of received (AC, numerator) light, which has penetrated the artery at a certain point in time, to incident light (DC, denominator).

(7) $$\begin{array}{c} T_{\lambda ,a}\left(t\right)=\frac{I_{\lambda }^{t}}{I_{\lambda }^{ia}}=\frac{\left(I_{\lambda }^{i}-I_{\lambda }^{r}\right)\cdot e^{-(\alpha \cdot l+\alpha_{a}\cdot l_{a}\left(t\right))}}{\left(I_{\lambda }^{i}-I_{\lambda }^{r}\right)\cdot e^{-\alpha_{a}\cdot l}}=e^{-\alpha_{a}\cdot l_{a}\left(t\right)} \end{array}$$

Consequently, the transmission ratio $\tau_{\lambda }$ can be calculated from the AC (numerator) and the DC (denominator) value.

(8) $$\begin{array}{c} \tau_{\lambda }\left(t\right):=\ln\left(e^{-\alpha_{a}\cdot l_{a}\left(t\right)}\right)=\alpha_{a}\cdot l_{a}\left(t\right) \end{array}$$

Using two different wavelengths and the ratios, the definition of the so-called ratio of ratio *R* is possible to be calculated directly out of the measurement by applying the logarithm.

(9) $$\begin{array}{c} R:=\frac{\tau_{1,a}}{\tau_{2,a}} \end{array}$$

The relative saturation *S* describes the ratio of oxyhemoglobin $c_{HbO_{2}}$ to total hemoglobin $c_{HbO_{2}}+c_{HbO}$.

(10) $$\begin{array}{c} S:=\frac{c_{HbO_{2}}}{c_{HbO_{2}}+c_{HbO}} \end{array}$$

The ratio of ratio can also be described by the extinction coefficients and the relative saturation of the blood, see Equation (11).

(11) $$\begin{array}{c} R:=\frac{\varepsilon_{HbO_{2},\lambda_{1}}\cdot S+\varepsilon_{Hb,\lambda_{1}}\cdot \left(1-S\right)+E_{others,\lambda_{1}}}{\varepsilon_{HbO_{2},\lambda_{2}}\cdot S+\varepsilon_{Hb,\lambda_{2}}\cdot \left(1-S\right)+E_{others,\lambda_{2}}} \end{array}$$

If now converted to saturation, it is possible to calculate the relative saturation *S* including *R* as described previously.

(12) $$\begin{array}{c} S\approx \frac{\left(R\cdot \varepsilon_{Hb,\lambda_{2}}-\varepsilon_{Hb,\lambda_{1}}\right)}{\left(\varepsilon_{HbO_{2},\lambda_{1}}-\varepsilon_{Hb,\lambda_{1}}\right)-R\cdot \left(\varepsilon_{HbO_{2},\lambda_{2}}-\varepsilon_{Hb,\lambda_{2}}\right)} \end{array}$$

Since Equation (12) is a simplification which does not take into account all the constituents of the blood, this formula should be considered only as an approximation. In practice, the measurement system must be calibrated or use more wavelengths to resolve more components [20,22,23,24,25].

### 2.2. Signal Processing

The implementation of a camera-based method of pulse oximetry requires various considerations in reference to the physical conditions, mathematical methods and resulting signal processing, which will be reviewed below. Commercial PO-meters are contact-based, which means that both the transmitter’s light-emitting diodes (LEDs) and the receiver unit are located directly on the skin. For newborns, the foot, palm or wrist is usually used, as fingers are too small for correct probe placement [26,27]. A circular bandage ensures that the probe is fastened securely in place and simultaneously forms a shield against extraneous light. The transmitter and receiver are located opposite one other to allow measurement through transmission.

The developed method of camera-based contactless pulse oximetry shows some significant differences compared to the conventional method. The following physical differences have to be considered: The light source and light-sensitive receiver are located next to each other and on one side of the object, so that the reflection and transmission is measured [28]. The distance between the sensor and the object of interest is variable and generally further away when compared to that of the commercial PO-meter. The vital parameters of premature infants must be monitored continuously in an incubator. These incubators set a distance of approximately 0.3 m between the neonate and the sensor, which is located on top of the incubator.

Various methods use passive ambient light as a source and realize the differentiation of wavelengths by spectral filters, for example an RGB Bayer filter or two cameras [25,29,30,31,32,33]. The method presented here, however, uses active lighting, which guarantees constant illumination.

**Figure 3:**
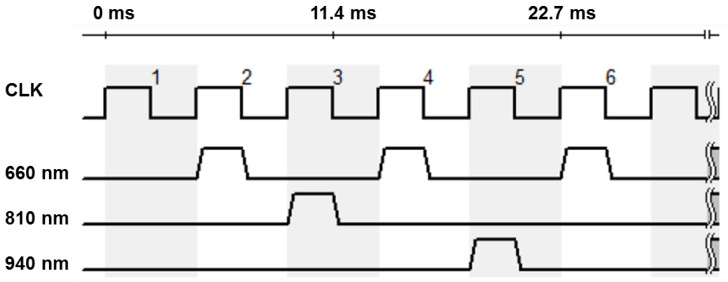
Time diagram of the LEDs for standard settings. The numbers indicate the clock cycles. During the first clock cycle, there is an empty measurement without any illumination; during the second cycle, the 660 nm LEDs are switched on; during the third cycle, the 810 nm LEDs; during the fourth cycle, the 660 nm LEDs again; during the fifth cycle, the 940 nm LEDs; and during the sixth cycle, the 660 nm LEDs again. Then the sequence repeats from the beginning.

The involved monochrome camera (IDS Imaging Development Systems GmbH, Obersulm, Germany) does not allow any spectral distinctions. In order to achieve a spectral resolution, the lighting is operated in a synchronous time-division multiplex process. The three available types of LEDs are operated alternately. In the following considerations and evaluations, the multiplex scheme shown in Figure 3 is preferred. The sampling rate of the camera is decisive for the selection of the sequence. For the intended application in the incubator, it is important that the light emitted does not flicker visibly, as this is unpleasant and can cause distress in premature infants.

At the selected area of interest (AOI) of 250 × 250 px, the maximum frame rate of the camera is about 220 fps. This results in an effective sampling rate of 110 Hz for a given multiplex time scheme for 660 nm, so that the strobe light is no longer visible to the human eye. In addition, sample 1 is without any illumination in order to subtract the background light from all the other measurements at each wavelength under the assumption that the background light varies slightly.

All other wavelengths alternate at approximately 36 Hz. This illumination of the measuring object must be strong enough to ensure sufficient signal strength and quality as well as dominate over extraneous background (bg) light. The incubator is not shielded from ambient light. The following condition applies:

(13) $$\begin{array}{c} I_{\lambda }^{i}\gg I_{bg} \end{array}$$

If this condition is fulfilled, a large part of the light radiating to the skin is reflected (remission, $I_{\lambda }^{r}$). The light penetrating the skin is attenuated, penetrates arteries and is captured as a signal in the camera/receiver when it leaves the skin. Thus, a combination of transmission and reflection is measured, see Equations (15) and 16. With these properties, the system is described as a time-dependent signal $x_{\lambda }\left(t\right)$ and background light $x_{bg}$.

(14) $$x_{\lambda }\left(t\right)=\left[\begin{array}{c} x_{660}\left(t\right)\\ x_{810}\left(t\right)\\ x_{940}\left(t\right) \end{array}\right];x_{bg}$$

The reflected and transmitted intensity $I_{\lambda }^{r}$ and $I_{\lambda }^{t}$ shown in Figure 2 can be described by the reflection factor $\Gamma_{\lambda }$ and the transmission factor $T_{\lambda }$, too.

(15) $$T_{\lambda }=\frac{I_{\lambda }^{t}}{I_{\lambda }^{i}}$$

and

(16) $$\Gamma_{\lambda }=I_{\lambda }^{i}-T_{\lambda }$$

It is to be assumed that $I_{\lambda }^{r}$ is constant. This supposition describes the phenomenon in which reflected light behaves independently of the pulse wave and thus independently of the time. The recorded signal can therefore be described as follows:

(17) $$x_{\lambda ,bg}=I_{\lambda }^{i}\left(t\right)\cdot \Gamma_{\lambda }\cdot T_{\lambda }\left(t\right)+x_{bg}$$

The signal term influenced by the background light can be separated into two varying parts, one part called $x_{bg-}$, influenced by the time-varying transmission of a static background light, and one signal part called $x_{bg∼}$ from a time-varying background light with constant background illumination.

(18) $$x_{bg}=\underset{x_{bg-}}{\underbrace{I_{bg}^{i}\cdot \Gamma_{bg}\cdot T_{bg}\left(t\right)}}+\underset{x_{bg∼}}{\underbrace{I_{bg}^{i}\left(t\right)\cdot \Gamma_{bg}\cdot T_{bg}}}$$

It is obvious that the useful signal is strongly influenced by the background light. The active illumination is presumed to be constant in amplitude, otherwise a further modulation is added which falsifies the measurement. As shown in Figure 3, one frame per multiplex sequence is a zero frame when the light source is switched off. It is utilized to reduce the influence of background light. The constant included in the “bg”-calculation can be reduced by subtracting the zero image as long as it remains unchanged over time. It follows

(19) $$\begin{array}{c} x_{\lambda }=x_{\lambda ,bg}-x_{bg} \end{array}$$

**Figure 4:**
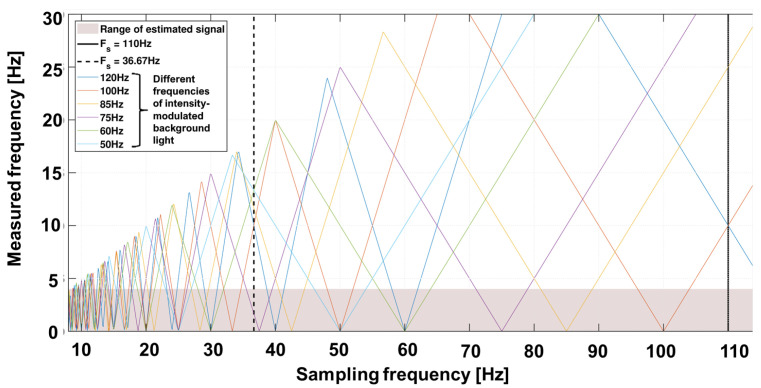
Undersampling diagram for expected background light frequencies over different sampling frequencies. Example: A 50 Hz modulated light (light blue color) results in 10 Hz (maximum) when sampled with 20 Hz, in 0 Hz when sampled with 25 Hz and in 16.6 Hz when sampled with 33.3 Hz. The selected sampling frequencies are chosen for 660 nm at 110 Hz (black line) and 810/940 nm at 36.67 Hz = 110/3 Hz in a manner that the undersampling frequencies of the modulated background light are out of the frequencies of interest.

The superimposed signal $x_{bg}$ now consists of the time-varying signal background [20,23,24,25]. The frequencies to be expected are above the useful signal band. However, the previously described sampling rate of the camera leads to sub-sampling and thus allows for aliasing. The expected external sources, including the main frequency at 50 Hz, tubes at 100 Hz or screens at 75 Hz, can lie within the useful bandwidth. Figure 4 shows an example of the spectrum to be expected with various background artifacts. The variability of frame rate allows a suitable selection in which the useful bandwidth contains no or as few artifacts as possible.

**Figure 5:**
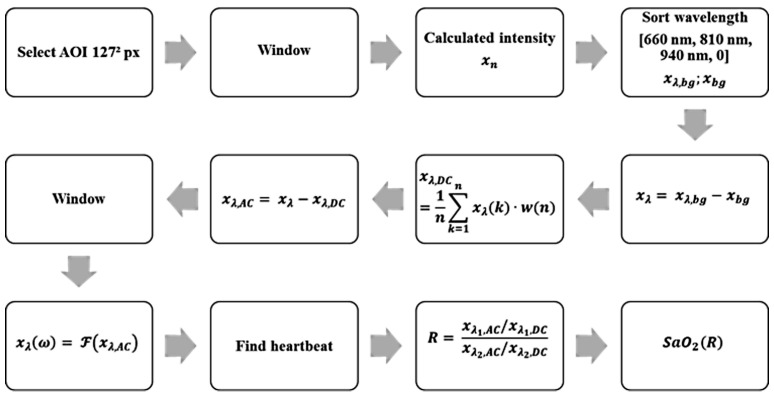
Block diagram of the algorithm used for the detection of a heartbeat and calculation of the oxygen saturation.

Using these parameters and properties, an algorithm for the detection of a heartbeat and calculation of the oxygen saturation was designed. The block diagram in Figure 5 shows the algorithm, explained below:

Display a default image of the recorded scene, in which a suitable place for the sub-AOI (125 px)² is selected.
Rework each image with a 2D Hann window to filter light movement. The Hann window is parameterized in a manner that the window function obtains zero at the edge of the window with $L_{Hann}=125$ px. Pixels close to the edges are weighted less than in the center.
Perform weighted averaging of all pixels based on the 2D Hann window to one intensity value. Thus, the given quantization is increased from 10 bits by 7 bits $\sqrt{125^{2}}=125\approx 2^{7}$ to 17 bits (compare the oversampling effect) in amplitude. Due to the effect that the quantization introduces uniformly distributed noise with a level of $\pm 0.5/2^{10}$, this noise is reduced by averaging under the assumption that the noise in each pixel is independent from other pixels. The variations due to heartbeat are comparable in each pixel; thus, averaging does not influence the level of the heartbeat signal. Without this virtual increase, the heartbeat signal cannot be reliably detected because it is in the range of the noise level.
Sort the discrete time values to match the corresponding spectral component.
Subtract the black value from each signal to remove constant background lighting.
(A) Average the weighted window of a length of 12 s to obtain DC.
  (B) Subtract the DC value in the current time window and thus determine the AC part of the signal.
Adjust spectral components in the phase by multiplying with $e^{-\Delta \Phi n}$, where ΔΦ corresponds to 2πfpsΔt and n is the corresponding timestamp in each multiplex phase starting with zero.
Rework the last 12 s of the signal with a window function to obtain a periodic signal.
Transform each window (12 s) from the time domain into the frequency domain by a fast Fourier transformation including zero padding to obtain a length of 8192 points.
Classify the pulse signal in $x_{\lambda }\left(\omega \right)$.
Calculate the ratio of ratio.
Calculate oxygen saturation according to Equation (12).

Steps one through seven are repeated for each multiplex phase, and steps eight through twelve are repeated every 22 multiplex phases to reduce computational effort.

### 2.3. Camera

In order to continuously improve these algorithms, the development of a modular and dynamic system was necessary. The most important components are the cameras, which were selected based on different parameters. These include resolution, quantization, quantum efficiency and the possibility of communication/software integration. Two cameras were used for the described system, a monochrome UI-3240ML-NIR-GL and an RGB camera UI-3240ML-M-GL from IDS Imaging Development Systems GmbH, Obersulm, Germany. Both have the option of multi-AOI, which makes it possible to increase the sampling frequency when reducing the recorded scene. This provides advantages in signal processing, such as an increase in 10-bit quantization, as described in Section 2.2.

Both sensors are equipped with redundant electronics so that they can be operated as master or slave. The master is used to record the vital data, while the slave can be used to synchronously record the scene to identify artifacts such as movements during an offline evaluation. Both cameras have a USB 3.0 port for communication and data transfer as well as general purpose input/output (GPIO) pins to control the illumination electronic.

### 2.4. Microcontroller and Illumination

The GPIO outputs of the master control an STM8 microcontroller (μC) of the driver board by means of a software SPI. It is not mandatory that the camera serves as a clock; it is also possible to use the μC as a clock source. The board also has GPIOs, which allows stacking of any number of these drivers to increase the light output.

**Figure 6:**
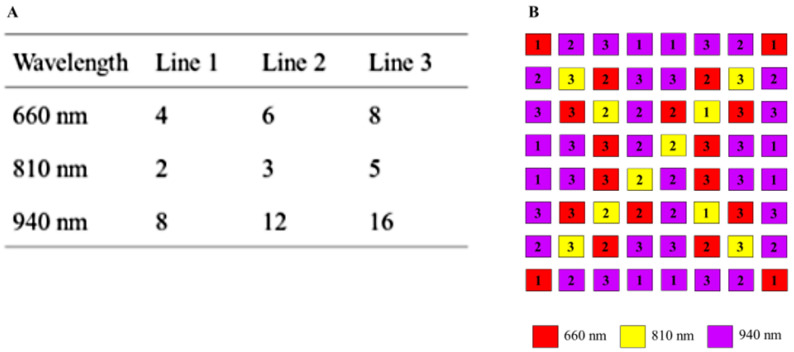
(**A**) Number of LEDs per supply line and wavelength. (**B**) Arrangement of LEDs for lighting board. Numbers indicate the supply lines.

Variants of the multiplex methods are predefined in the software and can be freely selected. In addition to switching on additional lighting boards, the intensity can be adjusted by switching three different supply lines with LEDs for each wavelength on and off. The intensity of the illumination is set for each wavelength, where the maximum value (white image) is not exceeded at a certain exposure time. This increases the signal-to-noise ratio. Figure 6A shows the number of LEDs per wavelength and per respective supply line. The pattern of each supply line is normally distributed so that a homogeneous illumination is achieved. Thus, up to seven intensity states are possible by combining the supply lines in a binary manner. The number of LEDs per wavelength compensates the spectral quantum efficiency of the sensor. Figure 6B shows the arrangement of the illumination board. At a distance of 0.3 m between ceiling and floor of the incubator, even a bright field illumination is guaranteed. The size of the LED panel is 4.0 cm × 3.1 cm.

**Figure 7:**
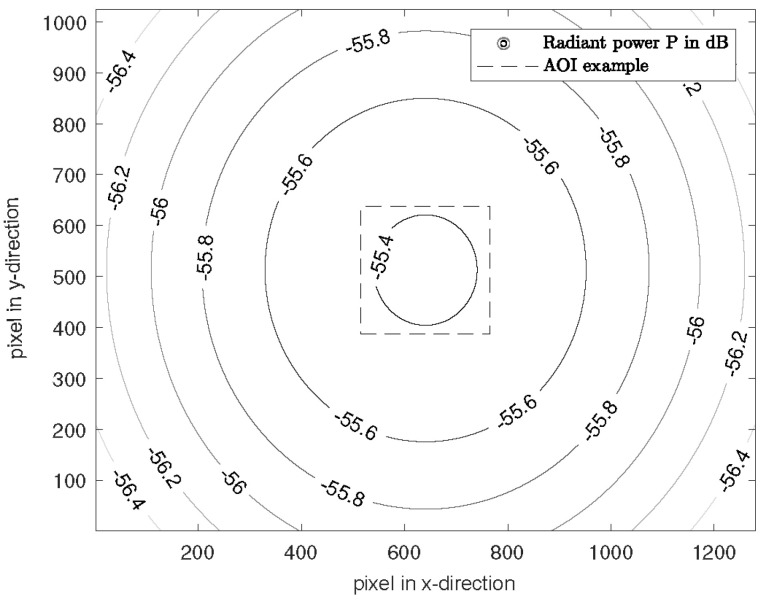
Attenuation normalized to the intensity $I_{\lambda }^{i}$ for the wavelength of 940 nm on the target. The pixel pitch and pixel size is 5.3 μm.

Figure 7 shows the simulated radiant power for the 940 nm LEDs on a plane object as an example. For other wavelengths, the distribution is similar, with different attenuation caused by the number of LEDs.

The minimum attenuation for 660 nm is −58.98 dB, for 810 nm −60.93 dB and for 940 nm −55.37 dB. In the entire scene (1280 × 1024 px), the maximum attenuation difference is 1.9 dB, so that diffuse lighting is created.

After setting up all of the hardware components, a storage system for the raw data was required, as the data evaluation was to be carried out offline. Furthermore, due to the high data rates in the range of 26.3 MByte/s, caused by the sampling rates and resolutions, online data evaluation including storage is currently not possible. The software comprises two programs: First, the “Record GUI”, which handles all camera settings and data storage, and the second, “Analysis GUI”, for offline evaluation.

“Record GUI” uses a USB 3.0 standard to set the camera, which in turn uses a software SPI to set the lighting unit. For an optimal adjustment of the measurement area, it is necessary to view the entire scene in which the AOI to be examined is placed at (250 px)^2^ before starting the recording. The whole AOI is divided into four sub-AOIs with 125 × 125 px each; these are each freely selectable. Each window can be viewed live for analysis and evaluation of the extraneous light. Thus, artifacts and their frequencies are detectable at an early stage. To reduce the artifacts in the useful signal bandwidth, see Section 2.2; the frame rate is adjusted. The optimal exposure is selected by switching LEDs on or off. Recording begins with the settings selected by the multiplex method.

At the beginning of analysis, a sub-AOI (125px)² is selected on an illuminated preview image. These windows as well as the data of the reference monitor (CARESCAPE B650, GE Healthcare, Chicago, IL, USA) are synchronized, processed and evaluated as described in Section 2.2. The values of the heartbeat and SpO_2_ are then stored alongside the comparison data from the reference monitor.

## 3. Experimental Setup

The various hardware components described above were integrated into an Incubator 8000 IC (Drägerwerk AG & Co KGaA, Lübeck, Germany). To evaluate the camera-based contactless pulse oximeter with time-division multiplex lighting as a novel tool in the NICU, a series of measurements were carried out. The vital parameters data should correspond as closely as possible to those of the later target group, premature infants. Similar to human preterm neonates, newborn piglets have a heart rate that ranges between 100 and 180 bpm [34]. For this reason, the measurement series was carried out on nine piglets aged between one and four weeks.

In preparation for the experiments, a pregnant sow (Aachen Minipig, Heinrichs Tierzucht GmbH, Heinsberg, Germany) was housed in the animal husbandry department bay with special litter three weeks before the expected date of delivery. After spontaneous birth, the weaning piglets had constant access to water and a warming lamp. Nine piglets (Aachen Minipig) from one litter were included in the study at the age of one to four weeks. Sedation was induced with 4% isoflurane in oxygen via a face mask to place a peripheral venous catheter in one of the ear veins. Blood was drawn from the catheterized vein through a three-way stopcock for blood gas analysis. Anesthetic maintenance was achieved by anesthetic depth-controlled IV administration of propofol (2–3 mg/kg/h). In order to reduce movement artifacts and to detect solely breathing and heart rate, this moderate sedation of the piglets was necessary. The spontaneously breathing piglet was placed in the center of the temperature-regulated incubator. This study was carried out in compliance with ARRIVE guidelines (Animal Research: Reporting of In Vivo Experiments [35]). All measurements were taken under constant supervision of physicians and veterinarians.

Data collection for each piglet involved four to five separate measurements in one day. During a period of 5 min to 25 min, the piglet was simultaneously monitored using both the contactless camera-based method described above and a conventional wired NICU monitor. To ensure optimal signals, we tested the contactless monitoring system using different locations of the sensor.

**Figure 8:**
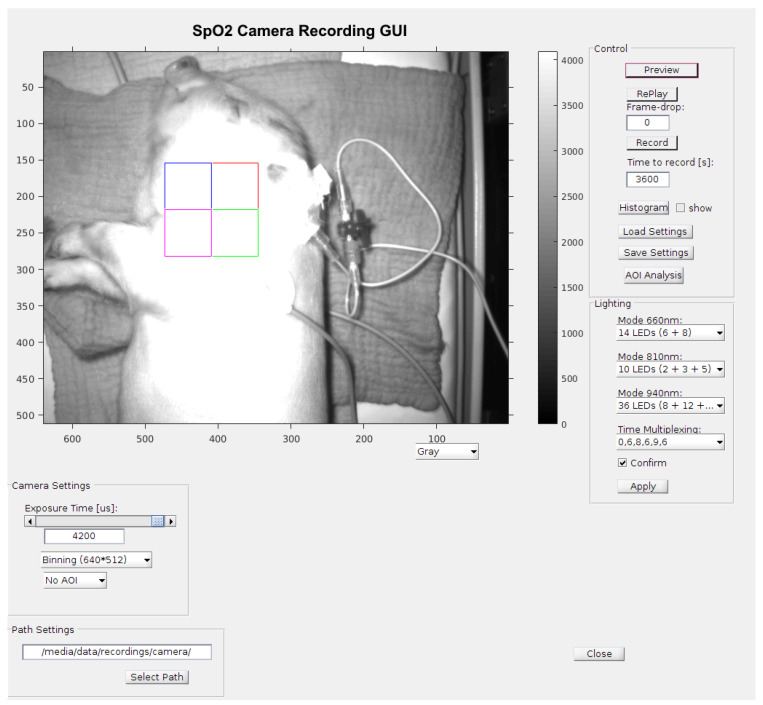
A screenshot (2) Figure seem to be cut on the top in a way that may affect scientific reading. Please check and provide whole image. of the measurement GUI including a picture of the piglet in the incubator with active illumination. The quadratic area is the area of interest (AOI) of 128 × 128 pixels for signal processing. The colors of the quadratic sub-areas have no additional meaning.

**Figure 9:**
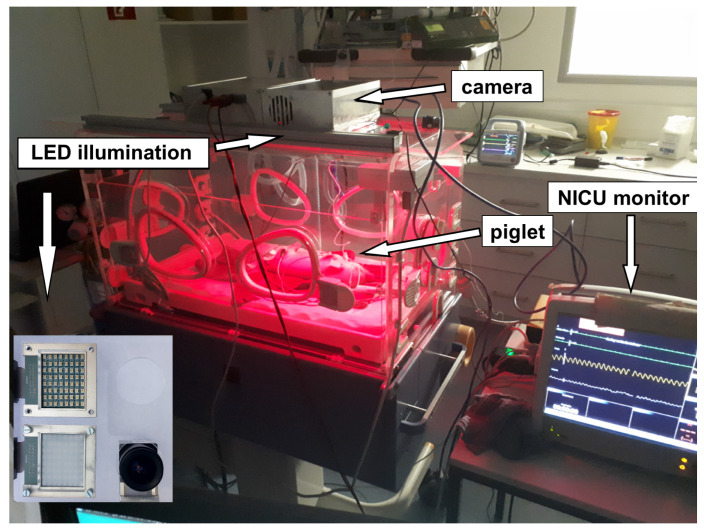
Measurement setup: A piglet was placed in a NICU incubator. Pulse, oxygen saturation and respiration rate were measured by a camera-based contactless pulse oximeter which was placed on the incubator. For validation, an independent monitor, which is commonly used in neonatal intensive care units, was linked via a pulse oximeter sensor to the piglet leg together with a three-channel electrocardiograph. The red color in the incubator is caused by the active illumination of the SpO_2_ sensor system and not from an infrared heating lamp, which would disturb the measurement. The camera and the illumination are mounted at the bottom side of the metallic box above the incubator and point into the incubator. The camera and the illumination are shown in the lower-left corner of the picture.

The optimum conditions of the above-mentioned time-division multiplex method are measurements taken in a relatively darkened room, so that the condition of Equation (13) is fulfilled. Thus, the adjustment of the sampling rate was not necessary; the maximum rate was selected. With the multiplex procedure used, all other procedures can be reproduced. To validate the data collected by the contactless monitoring, the vital parameters were measured additionally by an independent monitor (GE healthcare CARESCAPE B650), which is commonly used in neonatal intensive care units. The monitor was connected to the piglet legs via a pulse oximeter sensor, which uses red and infrared light to measure oxygen saturation (SpO_2_), together and with a three-channel electrocardiograph (ECG). The following Figure 8 shows the graphical user interface for the measurements including a picture of a sedated piglet. Figure 9 shows the setup during measurements.

## 4. Results

Firstly, we generated an extensive number of data using the contactless monitoring system with a high variation in the parameters. Using this as a base, error detection and improvement was possible. Thus, measurement data can be provided in case of a subsequent change in the algorithm. During the evaluation of the contactless monitoring system, the skin pulse rate was first extracted and compared to the heart rate measured by the ECG, for reference, as this has a significant influence on the accuracy of the saturation reading. Here we present the selected data collected from measurements of a single piglet concerning the detection of the heartbeat, skin pulse rate, respiratory rate and oxygen saturation using contactless monitoring.

### 4.1. Skin Pulse Rate and Respiratory Rate

**Figure 10:**
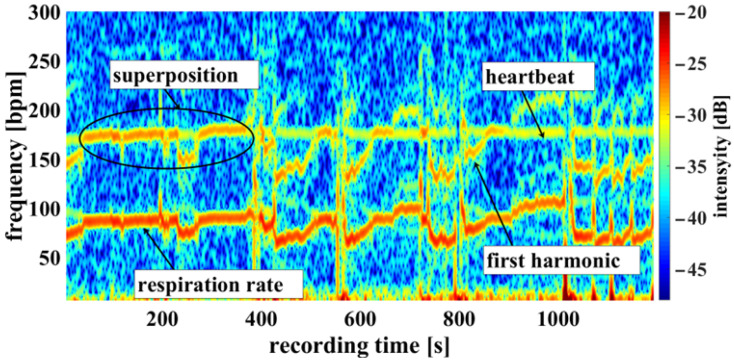
Example measurement 1. Camera data showing a recording from the oxygen saturation sensor: Example of a cumulated spectrum with respiration superimposed onto the heartbeat (multiplex scheme see Figure 3).

**Figure 11:**
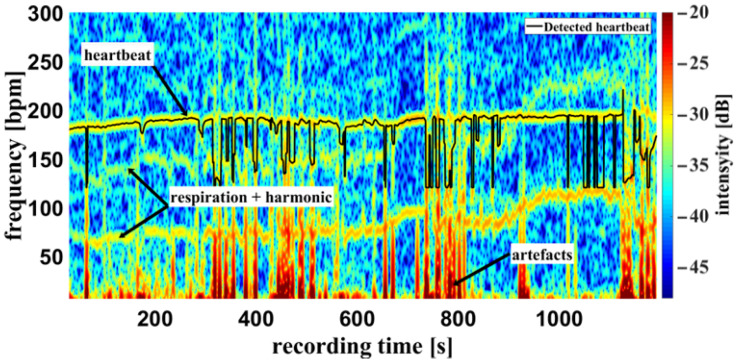
Example measurement 2. Camera data of a recording from the oxygen saturation sensor: Example of a cumulative spectrum with successful heartbeat detection (multiplex scheme see Figure 2). Heartbeat and respiration rate are clearly identifiable, as well as artifacts and respiratory interruptions.

Using the camera system, measurement of the respiratory rate was also possible. Some of the challenges encountered are demonstrated in Figure 10, an example of a cumulated spectrum. The respiratory rate of the piglet is around 90 bpm. Since variation due to respiration is a side effect of heartbeat measurement and the respiration signal is not an ideal sinus signal, the harmonics can superimpose upon the heartbeat. This may lead to a misinterpretation, depicted in Figure 10 between 0 and 400 s. So, it should be regarded as an unwanted artifact because it can lead to an incorrect measurement. It was also observed that the movements of the piglet led to modulation of light which can lie within the measuring bandwidth of the pulse wave, represented in Figure 11 around 800 s. The respiratory rate, skin pulse rate and pulse waves were recorded by contactless monitoring. Figure 11 shows a different measurement example of a cumulative spectrum with successful heartbeat detection. Heartbeat and respiration can be separated clearly during flat breathing and require only minor intervention, caused by artifacts and respiratory interruptions (mean difference: −9.193 bpm). The peaks, e.g., at 400 s and 700 s, are breathing interruptions.

### 4.2. Oxygen Saturation

**Figure 12:**
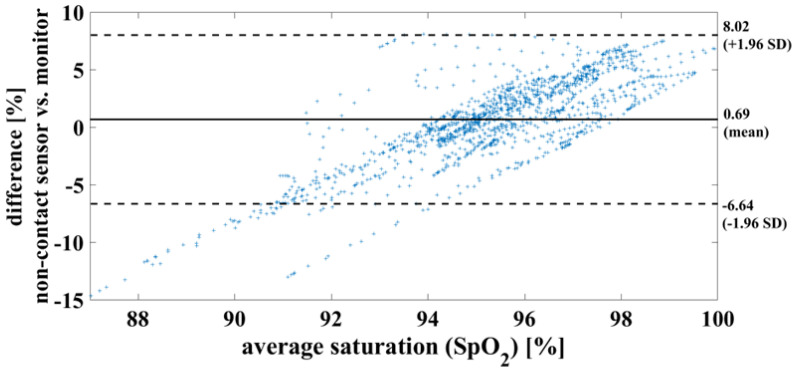
Bland– Altman diagram demonstrating the successful measurement of oxygen saturation of one piglet within a period of 20 min. This serves to compare the two measurement methods used.

In the case of a successful contactless-monitoring measurement, in which heartbeat and respiration can be clearly separated (Figure 11), the oxygen saturation can be determined with a mean difference of 0.7%, RMSE of 3.8% and MAE of 2.93%. To compare the results using the measurement methods, on the one hand contactless monitoring and on the other hand wired pulse oximetry, the results are shown here as a Bland–Altman diagram in Figure 12.

**Figure 13:**
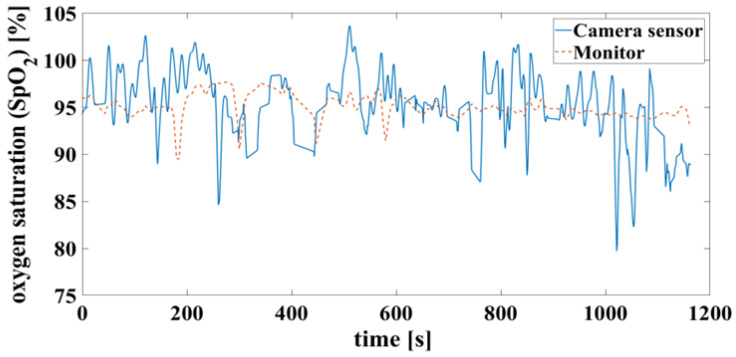
Time signals of oxygen saturation compare camera and monitor. Calculated according to Figure 11.

False detection of the heartbeat inevitably leads to an error in the oxygen saturation calculation. This can result in unrealistic saturation above 100%, shown in Figure 13, which is not yet filtered here.

## 5. Discussion

On average, every tenth child worldwide is born prematurely [1,2]. The enormous progress made in neonatological intensive care over the last two decades has significantly improved the survival prognosis for these children [36]. However, the very immature brain of the premature neonate is exposed to unphysiological stimuli and impairments through birth and the necessary intensive care measures [6,37].

This includes high susceptibility to infection [38], which increases proportionally with the number and intensity of medical interventions and manipulations. Irritations such as pain, noise and handling can lead to functional and structural aberrations during this vulnerable development phase of the brain [39]. By dispensing with the use of adhesive sensors, our newly developed contactless technology protects the sensitive skin of premature babies and thus reduces the risk of infection and the formation of lifelong scars [40,41]. The possible pain involved when applying and removing the sensors is also avoided [6]. It is important to seek to reduce stress factors for premature infants during their long stays in hospital and at the same time to improve the intensive medical treatment of these children.

For these reasons, an incubator that does not require wired sensors to record vital parameters is desired. This is intended allow a more “hands-off approach”, leading to the avoidance of the aforementioned stimuli and thus to the prevention of the harmful acute and long-term consequences for the premature infant. A first step in the development of this technology is the establishment of a camera-based contactless pulse oximeter that monitors the pulse rate, respiratory rate (error term) and oxygen saturation of the blood. The initial testing should determine the ability of the camera-based contactless pulse oximetry sensor to validly determine the pulse rate.

### 5.1. Skin Pulse Rate and Respiratory Rate

The conventional monitor used by default in the NICU served as a reference to validate the measurements based on the contactless monitoring system. Throughout all recorded measurements, multiple deviations of the conventional and contactless monitoring systems were observed during the experiments. Multiple factors contributed to the deviations between the two measurement techniques. Respiration measurements of the contactless system (costal as well as abdominal) were caused by turbulent and extreme respiration leading to strong movements of the pig body. These, and also dislocation of the reference electrodes, do not allow reliable measurements with conventional algorithms. These errors cannot be detected, which leads to mistakes in the algorithm. A further error is the frequency which is modulated by the breathing of the pig: breathing modulation is the result of changing the distance between the skin and the camera during a breathing cycle. Under the simplified assumption of two isotropic emitters (LED, skin), a distance (*r*) change modulates the light intensity by $1/r^{2}$. Furthermore, the observed spots on the piglet skin move laterally to the sensor, which results in a shift across the pixels and yields an intensity variation. This movement results in a noise-like signal in each pixel, avoiding a clear heartbeat detection.

However, newborns physiologically have periodic modulations of the respiration rate due to an immature respiratory center. Apneas of up to 20 s are regarded as physiological in term neonates unless they are followed by consecutive bradycardia. Apnea of prematurity (AOP), which is characterized by apneas with consecutive bradycardia, are common among preterm neonates. Detection of AOP needs the continuous and precise measurement of breathing movements, heart rate and arterial oxygen saturation. The conventional monitoring of vital parameters using the wired NICU monitor is also subject to considerable artifacts. Artifacts are detected and defined on the conventional monitor by the fact that the ECG waves do not clearly indicate an R tick and that the pulse oximeter indicates a different pulse rate than the heart rate measured by the ECG. For respiration, there is no reliable method to identify artifacts.

### 5.2. Oxygen Saturation

In a quiet, narcotized piglet, the contactless monitoring reliably yielded similar values for oxygen saturation as conventional pulse oximetry. However, the contactless measurement was subject to artifacts. For example, movements of the patient, pigmentation of the skin, poor blood circulation and room brightness limit accurate measurements [11,42,43,44,45,46].

It is important to detect respiration and separate it from the useful signal. Estimating the heartbeat and its signal amplitude is the key to calculating arterial saturation. In addition, clinical calibration is required to eliminate constant errors, which is currently not possible due to cost and effort. This step makes sense if all other sources of error have been eliminated in the best possible way.

## 6. Conclusions

In conclusion, we successfully developed a novel sensor for the contactless measurement of heart rate, respiration and oxygen saturation. This novel sensor combines a monochrome camera with a time-division multiplex controlled lighting with three different wavelengths in the red and infrared spectra (660 nm, 810 nm, and 940 nm). Our proof of principle experiments in a neonatal piglet yielded highly reliable results when compared to conventional monitoring in a calm, motionless subject.

In our sensor, the measurement of the oxygen saturation requires the optical detection of the heart rate and breathing. As a result, the measurements of oxygen saturation and heart rate were prone to artifacts when the subject was agitated, since movements of the whole body could interfere with the signals of breathing and heartbeat. Moreover, pigmentation of the skin, poor blood circulation and excessive environmental light can reduce the accuracy of the measurements. Further experiments and adjustments of the detection algorithm are underway to optimize the camera-based contactless measurement of vital signs in preterm neonates.

## Data Availability

The program code used in this study cannot be disclosed as it is proprietary to ELABS AG, Germany, and is subject to confidentiality agreements. The data utilized are owned by CUBICAL GmbH, Germany; they are not available for public disclosure. Both ELABS AG and CUBICAL GmbH were participants in the BMBF-funded project.

## Abbreviations

The following abbreviations are used in this manuscript:

AC	Alternating current; here, the time-varying signal part
AOI	Area of- nterest
AOP	Apnea of prematurity
bpm	Beats per minute
CLK	Clock
ECG	Electrocardiography
DC	Direct current; here, the time-constant signal part
dB	Decibel
GE	General Electric
GUI	Graphical user interface
GPIO	General purpose input output
Hb	Deoxyhemoglobin
HbO_2_	Oxyhemoglobin
IDS	Imaging Development Systems GmbH
LED	Light-emitting diode
MAE	Mean absolute error
μC	Microcontroller
NICU	Neonatal intensive care unit
PO	Pulse oximetry
px	Pixel
RGB	red–green–blue color space
RMSE	Root mean squared error
rPPG	Remote photoplethysmography
PPG	Photoplethysmography
SpO_2_	Oxygen saturation
USB	Universal serial bus

## References

[1] Purisch S.E., Gyamfi-Bannerman C. (2017). Epidemiology of preterm birth. Semin. Perinatol..

[2] Goedicke-Fritz S., Hartel C., Krasteva-Christ G., Kopp M.V., Meyer S., Zemlin M. (2017). Preterm Birth Affects the Risk of Developing Immune-Mediated Diseases. Front. Immunol..

[3] Glass H.C., Costarino A.T., Stayer S.A., Brett C.M., Cladis F., Davis P.J. (2015). Outcomes for extremely premature infants. Anesth. Analg..

[4] Chung H.U., Kim B.H., Lee J.Y., Lee J., Xie Z.Q., Ibler E.M., Lee K., Banks A., Jeong J.Y., Kim J. (2019). Binodal, wireless epidermal electronic systems with in-sensor analytics for neonatal intensive care. Science.

[5] Cartlidge P.H., Fox P.E., Rutter N. (1990). The scars of newborn intensive care. Early Hum. Dev..

[6] Ranger M., Grunau R.E. (2014). Early repetitive pain in preterm infants in relation to the developing brain. Pain Manag..

[7] Aoyagi T. (1974). Improvement of the earpiece oximeter. Abstracts of the Japanese Society of Medical Electronics and Biological Engineering.

[8] Poets C.F., Southall D.P. (1994). Noninvasive monitoring of oxygenation in infants and children: Practical considerations and areas of concern. Pediatrics.

[9] Aarts L.A., Jeanne V., Cleary J.P., Lieber C., Nelson J.S., Bambang Oetomo S., Verkruysse W. (2013). Non-contact heart rate monitoring utilizing camera photoplethysmography in the neonatal intensive care unit—A pilot study. Early Hum. Dev..

[10] Liu C., Correia R., Ballaji H.K., Korposh S., Hayes-Gill B.R., Morgan S.P. (2018). Optical Fibre-Based Pulse Oximetry Sensor with Contact Force Detection. Sensors.

[11] Barker S.J. (2002). ”Motion-resistant” pulse oximetry: A comparison of new and old models. Anesth. Analg..

[12] Grooby E., Sitaula C., Chang K.T., Sharkey D., Marzbanrad F., Malhotra A. (2023). Artificial intelligence-driven wearable technologies for neonatal cardiorespiratory monitoring: Part 1 wearable technology. Pediatr. Res..

[13] Sivanandan S. (2023). Wearables—A Revolution in Neonatal Monitoring?. Indian J. Pediatr..

[14] Zhou L., Guess M., Kim K.R., Yeo W.-H. (2024). Skin-interfacing wearable biosensors for smart health monitoring of infants and neonates. Commun. Mater..

[15] Pirzada P., Wilde A., Harris-Birtill D. (2024). Remote Photoplethysmography for Heart Rate and Blood Oxygenation Measurement: A Review. IEEE Sensors J..

[16] Svoboda L., Sperrhake J., Nisser M., Taphorn L., Proquitté H. (2024). Contactless assessment of heart rate in neonates within a clinical environment using imaging photoplethysmography. Front. Pediatr..

[17] Svoboda L., Sperrhake J., Nisser M., Zhang C., Notni G., Proquitté H. (2022). Contactless heart rate measurement in newborn infants using a multimodal 3D camera system. Front. Pediatr..

[18] Prahl S. Tabulated Molar Extinction Coefficient for Hemoglobin in Water. https://omlc.org/spectra/hemoglobin/summary.html.

[19] Wieringa F.P., Mastik F., van der Steen A.F. (2005). Contactless multiple wavelength photoplethysmographic imaging: A first step toward ”SpO_2_ camera” technology. Ann. Biomed. Eng..

[20] Aoyagi T. (2003). Pulse oximetry: Its invention, theory, and future. J. Anesth..

[21] Severinghaus J.W. (2007). Takuo Aoyagi: Discovery of pulse oximetry. Anesth. Analg..

[22] Verkruysse W., Bartula M., Bresch E., Rocque M., Meftah M., Kirenko I. (2017). Calibration of Contactless Pulse Oximetry. Anesth. Analg..

[23] Humphreys K.G. (2007). An Investigation of Remote Non-Contact Photoplethysmography and Pulse Oximetry. Ph.D. Thesis.

[24] Hülsbusch M. (2008). Ein bildgestüTztes, Funktionelles Verfahren zur Optoelektronischen Erfassung der Hautperfusion. Ph.D. Thesis.

[25] Wieringa F.P. (2007). Pulse Oxigraphy: And Other New In-Depth Perspectives through the Near Infrared Window. Ph.D. Thesis.

[26] Phattraprayoon N., Sardesai S., Durand M., Ramanathan R. (2012). Accuracy of pulse oximeter readings from probe placement on newborn wrist and ankle. J. Perinatol..

[27] Safar H., El-dash H. (2015). Pulse Oximetry: Could Wrist and Ankle Be Alternative Placement Sites?. Clin. Pediatr..

[28] Nijland R., Nierlich S., Jongsma H.W., Nijhuis J.G., Oeseburg B., Springer K., Mannheimer P. (1997). Validation of reflectance pulse oximetry: An evaluation of a new sensor in piglets. J. Clin. Monit..

[29] Tarassenko L., Villarroel M., Guazzi A., Jorge J., Clifton D.A., Pugh C. (2014). Non-contact video-based vital sign monitoring using ambient light and auto-regressive models. Physiol. Meas..

[30] Sun Y., Papin C., Azorin-Peris V., Kalawsky R., Greenwald S., Hu S. (2012). Use of ambient light in remote photoplethysmographic systems: Comparison between a high-performance camera and a low-cost webcam. J. Biomed. Opt..

[31] Kong L., Zhao Y., Dong Y., Jian Y., Jin X., Li B., Feng Y., Liu M., Liu X., Wu H. (2013). Non-contact detection of oxygen saturation based on visible light imaging device using ambient light. Opt. Express.

[32] Karlen W., Lim J., Ansermino J.M., Dumont G., Scheffer C. Design challenges for camera oximetry on a mobile phone. Proceedings of the 2012 Annual International Conference of the IEEE Engineering in Medicine and Biology Society.

[33] Kellicut D.C., Weiswasser J., Arora S., Freeman J., Lew R., Shuman C., Mansfield J., Sidawy A. (2004). Emerging technology: Hyperspectral imaging. Perspect. Vasc. Surg. Endovasc. Ther..

[34] Eisenhauer C.L., Matsuda L.S., Uyehara C. (1994). Normal physiologic values of neonatal pigs and the effects of isoflurane and pentobarbital anesthesia. Lab. Anim. Sci..

[35] Du Sert P.N., Ahluwalia A., Alam S., Avey M.T., Baker M., Browne W.J., Clark A., Cuthill I.C., Dirnagl U., Emerson M. (2020). Reporting animal research: Explanation and elaboration for the ARRIVE guidelines 2.0. PLoS Biol..

[36] Voigt M., Wittwer-Backofen U., Scholz R., Schneider K.T., Straube S., Olbertz D., Hesse V., Rochow N. (2013). Analysis of the German perinatal survey of the years 2007–2011 and comparison with data from 1995–1997: Neonatal characteristics and duration of pregnancy. Z. Geburtshilfe Neonatol..

[37] Brew N., Walker D., Wong F.Y. (2014). Cerebral vascular regulation and brain injury in preterm infants. Am. J. Physiol. Regul. Integr. Comp. Physiol..

[38] Chau V., McFadden D.E., Poskitt K.J., Miller S.P. (2014). Chorioamnionitis in the pathogenesis of brain injury in preterm infants. Clin. Perinatol..

[39] Bonan K.C., Pimentel Filho J.C., Tristao R.M., Jesus J.A., Campos Junior D. (2015). Sleep deprivation, pain and prematurity: A review study. Arq. Neuropsiquiatr..

[40] Onyeama C.O., Srinivasan H., Lotke M., Vickers D.L. (2009). Subgaleal abscess and *E. coli* septicemia following scalp electrode in a preterm newborn: A case report. J. Mater. Fetal Neonatal. Med..

[41] Rogdo B., Kahlert C., Diener P.A., Micallef J. (2014). Primary cutaneous aspergillosis in a preterm neonate. BMJ Case Rep..

[42] Langton J.A., Hanning C.D. (1990). Effect of motion artefact on pulse oximeters: Evaluation of four instruments and finger probes. Br. J. Anaesth..

[43] Severinghaus J.W., Spellman M.J. (1990). Pulse oximeter failure thresholds in hypotension and vasoconstriction. Anesthesiology.

[44] Villanueva R., Bell C., Kain Z.N., Colingo K.A. (1999). Effect of peripheral perfusion on accuracy of pulse oximetry in children. J. Clin. Anesth..

[45] Adler J.N., Hughes L.A., Vivilecchia R., Camargo C.A. (1998). Effect of skin pigmentation on pulse oximetry accuracy in the emergency department. Acad. Emerg. Med..

[46] Amar D., Neidzwski J., Wald A., Finck A.D. (1989). Fluorescent light interferes with pulse oximetry. J. Clin. Monit..

